# Trending Occupational Fatalities and Injuries: An Assessment of Projected Climate Change Related Impacts in the United States since 1992

**DOI:** 10.3390/ijerph20136258

**Published:** 2023-06-30

**Authors:** Charmaine Mullins-Jaime

**Affiliations:** Department of Built Environment, Bailey College of Engineering & Technology, Indiana State University, Terre Haute, IN 47809, USA; charmaine.mullins-jaime@indstate.edu

**Keywords:** effects of climate change, occupational fatalities, injuries, and illnesses, occupational illness and injury prevention

## Abstract

Background: Some impacts of climate change that are expected to affect the American workforce are rising temperatures, greater prevalence of wildland fires, increase in Lyme disease, and exposure to insecticides. The purpose of this study was to assess how fatal and non-fatal occupational injuries due to environmental heat, forest/brush fires, Lyme disease, and exposure to insecticides have changed over time in the United States and if there were any significant relationships between national occupational injury/illness data and national temperature trends. Methods: Linear regression models assessed fatal and non-fatal injuries/illnesses since 1992 by both the frequency of incidents and the proportion of total incidents and the effects of national average temperatures. Results: There were significant increases in occupational fatalities and illnesses due to exposure to environmental heat and national average annual temperatures were predictive of heat exposure fatalities and illnesses. Conclusion: Heat exposure is an occupational hazard that must be managed carefully in the coming years. Organizations will need to take more aggressive heat exposure control measures as temperatures continue to rise and remain hotter for longer periods during the year. While not currently showing increasing trends on a national scale, the prevalence of occupational incidents due to forest/brush fires, Lyme disease, and insecticides should be monitored as the United States experiences more of the projected impacts of climate change.

## 1. Introduction

Climate change and climate change-related impacts are diverse and often interconnected. There is a broad range of projected impacts in the United States (U.S.) including higher incidence of extreme heat, temperature extremes, hazardous weather events, drought, flooding, harsher growing conditions for food production, and higher prevalence of certain diseases including vector-borne diseases [1]. 

In addition to affecting the general public, there is a concern for climate change affecting occupational health and the productivity of the American workforce [2]. According to the George Washington University Milken Institute School of Public Health [3], International Labour Organization [4], and Oxford Research Encyclopedia of Global Public Health [5], some of the projected climate change impacts that are expected to affect worker health and safety are heat, ozone, pathogens, infectious diseases, polycyclic aromatic hydrocarbons, wildfires, and workplace violence. According to the U.S. Environmental Protection Agency (EPA), climate change-related threats to worker health in the United States are heat illness, respiratory illness, physical and mental effects mainly to do with climate change-related disasters (physical trauma and mental health effects such as anxiety, depression, and post-traumatic stress disorder), insect and tick-related diseases (such as Lyme disease, Zika, and West Nile Virus), and pesticide-related effects [6]. 

While much of the discussion around climate change is on future impacts, we are already beginning to see the effects of climate change such as a rise in annual average temperature in the U.S. [7] and globally [8], and a higher prevalence of severe weather events, changing weather patterns, and wildland fires [1,9]. From an occupational health and safety management perspective, it is reasonable to expect that people in the United States would already be impacted by some of the effects of climate change through their work. However, a comprehensive national assessment of occupational injury and illness data linked to climate change-related impacts has not been undertaken. 

This paper focuses on assessing historical trends in occupational injuries and fatalities in the United States since 1992, based on the associated effects of climate change, as indicated by the EPA, and other sources as noted above. Fatal and nonfatal cases involving days away from work that may be linked to climate change-related impacts were assessed for significant trends over time. Relationships between national annual average temperatures and injury and fatality trends were also assessed. 

The occupational injuries and fatalities evaluated in this study are fatal occupational injuries and nonfatal occupational injuries involving days away from work by exposure or event categories: “exposure to environmental heat” and “forest or brush fire”; by nature of condition category: “Lyme disease” and by primary source: “insecticides”. These injuries and fatalities were assessed based on the earliest to the latest years in which the data are available which are from years 1992–2021 for the Census of Fatal Occupational Injuries (CFOI) data and 1992–2020 for the Survey of Occupational Illness and Injuries (SOII). 

The United States has experienced an increase in annual average temperature over the last 30 years in which most of the U.S. was warmer over this period [6]. When temperatures exceed 87 °F, individuals become vulnerable to heat stress [10]. When body temperature rises to 104 °F, this becomes a life-threatening emergency [11]. Thus, assessing any changes in injury and fatality data due to exposure to environmental heat is an important first step in defining the current and historical burden on occupational health. Evaluating the impacts of forest fires on occupational health due to the effects of smoke in distal areas is outside the scope of this study due to limitations in the availability of data. However, the direct effects of forest or brush fires are readily available in the CFOI and SOII datasets. Hotter temperatures can cause drying of forests and bushlands, making them more vulnerable to fires [1,12]. Thus, assessing any increasing trends in occupational fatalities and injuries directly due to forest or brush fires is important in this study as a greater prevalence of these types of fires has been linked to climate change.

Lyme disease is the most common vector-borne disease in the U.S. and is caused mainly by the bacterium Borrelia burgdorferi [13] from the bite of infected black-legged ticks. Black-legged ticks pose a risk to humans in warmer and wetter conditions [14,15]. Hotter and drier conditions pose less of a hazard from Lyme disease [14,15]. According to the National Oceanic and Atmospheric Administration (NOAA) U.S. Climate Normal data, the eastern two-thirds of the U.S. was wetter from 1991 to 2020 [7]. Combined with warmer temperatures, this makes ideal conditions for black-legged ticks to spread Lyme disease in a large population of the United States. Thus, assessing the burden of Lyme disease on occupational illness data is important. 

Global warming and climate change are expected to create conditions for weeds and pests to thrive and will affect the use of insecticides where workers may be exposed to higher quantities and different kinds of insecticides [16]. In addition to their use for crop management, workers may also have additional exposure using insect repellent on their skin and clothing as a prevention for vector-borne diseases. Thus, assessing if and how worker injuries/illnesses due to insecticides are changing is worthwhile. 

The purpose of this study was to assess how fatal and non-fatal occupational injuries, that have been projected as climate change impacts on occupational health, have changed over time in the United States. This study assessed if there were significant increases in certain occupational fatalities and injuries over time, and if there were any significant relationships between national fatalities and injuries and national temperature trends. More specifically, this work intended to answer the following questions:

1. Has there been a significant increase in national occupational fatalities and injuries involving days away from work due to exposure to environmental heat, forest or brush fire, Lyme disease, and/or from insecticides?

**H1.**  *There is a significant increase in national fatal occupational injuries over time from 1992 to 2021 in one or more of these exposures, nature, and source categories.*

**H2.**  *There is a significant increase in national non-fatal occupational injuries involving days away from work over time from 1992 to 2020 in one or more of these exposures, nature, and source categories.*

2. What is the effect of national average temperatures on national occupational fatalities and injuries involving days away from work due to exposure to environmental heat, forest or brush fire, Lyme disease, and/or from insecticides?

**H3.**  *Higher national average temperatures are predictive of higher national fatal occupational injuries from 1992 to 2021 in one or more of these exposures, nature, and source categories.*

**H4.**  *Higher national average temperatures are predictive of higher national non-fatal occupational injuries involving days away from work from 1992 to 2020 in one or more of these exposures, nature, and source categories.*

## 2. Materials and Methods

### 2.1. Data Sources

Data were collected on fatal and non-fatal occupational injuries from the United States Bureau of Labor Statistics (BLS) online profiles tool [17] and National Oceanic and Atmospheric Administration (NOAA) National Centers for Environmental Information, Climate at a Glance: National Time Series [18]. Due to changes in the Occupational Injury and Illness Classification (OIICs) manual and changes in data collection requirements over the years [19], separate searches for the years 1992–2002, 2003–2010, and 2011–2021 were conducted for fatal injury data in the CFOI. Separate searches for the years 1992–2001, 2002, 2003–2010, and 2011–2020 (the latest year with complete injury data available) were conducted for non-fatal occupational injuries resulting in days away from work in the SOII.

### 2.2. Data Collection

Data on fatal occupational injuries were searched and retrieved by “all ownerships” and exposure or event categories: “exposure to environmental heat” (code 321) for years 1992–2002, 2003–2010 (code 321) and (code 531) for years 2011–2021, “forest, brush or other outdoor fire” (code 512) for years 1992–2002 and 2003–2010 (code 512) and “forest or brush fire” (code 3160) for years 2011–2021 and by primary source: “insecticides” (code 065) for years 1992–2002 and years 2003–2010 (code 065) and (code 1550) for the years 2011–2020. Lyme disease data, as a nature of the condition in fatal occupational injuries and illnesses, are not available for search in the CFOI and were thus excluded from the analysis of fatal injury data. However, it is unlikely to have found any pattern in Lyme disease over time as the prevalence as a fatal condition is relatively low in the general population according to the Centers for Disease Control and Prevention with eleven cases of fatal Lyme carditis reported worldwide between 1985 and 2019 [20]. Data on insecticides as a primary source of fatal illness/injury are not available for the years 2002–2010, thus fatal injuries due to insecticides over 30 years could not be assessed. Data on total fatal occupational injuries/illnesses per year were also collected.

Data on non-fatal injuries and illnesses involving days away from work were searched and retrieved by “private industry” as the SFOII only began publishing national estimates for state and local government after the year 2008 and thus private industry is the only basis of comparison over the 29-year history of non-fatal injuries/illnesses. Data were searched by exposure or event category: “exposure to environmental heat” (code 321) for years 1992–2001, 2002, and 2003–2010 (code 321), and (code 531) for years 2011–2020, “forest, brush, or other outdoor fire” (code 512) for years 1992–2001, 2002 and 2003–2010 (code 512) and “forest or brush fire” (code 3160) for years 2011–2020; by nature of condition: “Lyme disease” (code 237) for years 1992–2001, 2002, and 2003–2010 (code 237), and “Lyme disease” (code 333) for years 2011–2020 and by primary source: “insecticides” (code 065) for years 1992–2001, 2002, 2003–2010 (code 065), and (code 1550) for the years 2011–2020. Data on total non-fatal occupational injuries/illnesses involving days away from work per year were also collected.

Data were collected on national annual temperature averages for the period 1992–2021 from National Oceanic and Atmospheric Administration (NOAA) National Centers for Environmental Information, Climate at a Glance: National Time Series [18]. 

While precipitation is a factor in the population growth of black-legged ticks [21], precipitation levels were not compared with the prevalence of Lyme disease-related injuries/illnesses as national precipitation ranges vary broadly with some regions of the country experiencing wetter conditions over the last 30 years and some regions experiencing much drier conditions over the last 30 years [7]. An assessment of precipitation levels and temperature as predictors of occupational illness due to Lyme disease is better suited at the state and local levels where inferences can be made. However, as noted above, this study intended to only evaluate trends from a national level. While some regions of the country have experienced cooler trends in the last 30 years, the majority of the country has experienced higher temperatures resulting in an overall upward trend in temperature. Thus, one can more reasonably make an inference of national average temperatures as a possible predictor of certain national occupational injuries/illnesses and fatalities versus precipitation and humidity. 

### 2.3. Analysis

Injury data were tabulated and the frequency of injuries/illnesses and total national injuries are shown. Assumption testing for linear regression was performed. Linear regression models, using IBM SPSS V28, assessed fatal injuries over the 30 years of available data and the non-fatal injury data over the 29 years of available data by both the frequency of incidents and the proportion of total incidents. Assessments based on proportional data were included in addition to assessments of frequency data because they adjust for the decrease in overall injuries and fatalities the U.S. has experienced since 1992. Thus, it can give perspective on the proportional size of the problems relative to total annual fatalities and injuries/illnesses. The effects of national temperature on fatal and non-fatal injuries/illnesses over these 30-year and 29-year periods were also assessed. 

## 3. Results

### 3.1. Descriptive Statistics

Table 1 shows the total number of fatal occupational injuries/illnesses, the total number of occupational fatalities involving environmental heat exposure and forest or brush fires for the years 1992–2021, the percentage of each type of fatality compared with total annual fatalities, and the national annual average temperature for each year. Table 2 shows the total number of non-fatal occupational injuries/illnesses due to exposure to environmental heat, forest or brush fires, Lyme disease, and insecticides from 1992 to 2020 and the percentage of each type of injury/illness compared with annual totals.

### 3.2. Regression Results

Regression analyses on relationships between injury data and time and temperture are presented in Table 3 and Table 4. Fatal injuries over the 30 years of available data and the non-fatal injury data over the 29 years of available data by the frequency of incidents are displayed in Table 3. and by the proportion of total incidents in Table 4. 

### 3.3. Exposure to Environmental Heat

There was a total of 999 occupational fatalities due to exposure to environmental heat from 1992 to 2021, Table 1. There were significant positive linear relationships between fatal occupational injuries due to exposure to environmental heat and time when assessed by both frequency of incidents (B = 0.723, *p* = 0.001) Table 3 and as a proportion of total incidents (B = 0.00019, *p* ≤ 0.001), Table 4. There were also significant positive linear relationships between fatal occupational injuries due to exposure to environmental heat and national average temperature when assessed by both the frequency of incidents (B = 0.4912, *p* = 0.024) and as a proportion of total annual occupational fatalities (B = 0.001, *p* ≤ 0.030).

There was a total of 75,577 occupational injuries/illnesses involving days away from work due to exposure to environmental heat, Table 2. There were no significant trends for non-fatal injuries/illnesses due to exposure to environmental heat involving days away from work over time and average temperatures when assessed by the frequency of occurrence. However, when evaluated as a proportion of total annual injuries/illnesses involving days away from work, there were statistically significant positive linear relationships over time (B = 0.000134, *p* ≤ 0.001), Table 3, and by annual average temperatures (B = 0.001, *p* ≤ 0.021), Table 4.

### 3.4. Forest or Brush Fires

There was a total of 216 occupational fatalities involving forest or brush fires from 1992 to 2021, Table 1. There was a significant negative linear relationship between fatal forest and brush fires and time when evaluated based on the frequency of incidents (B = −0.242, *p* = 0.024). However, when analyzed based on the proportion of total fatal injuries/illnesses, the downward trend was not statistically significant. National average temperatures were not predictive of fatal occupational injuries/illnesses involving forest and brush fires under both models. 

There was a total of 1015 cases of nonfatal occupational injuries/illnesses involving days away from work due to forest or brush fires from 1992 to 2020, Table 3. There were significant negative linear relationships between both the frequency (B = −3.491, *p* ≤ 0.001), Table 3, and proportion (B = −0.522, *p* = 0.004), Table 4, of non-fatal occupational injuries/illnesses involving days away from work due to forest and brush fires over time. There was also a significant negative linear relationship between the frequency of nonfatal occupational injuries/illnesses involving days away from work due to forest and brush fires and national average temperatures (B = −18.329, *p* = 0.039). However, there was no significant relationship between the proportion of these injuries and national average temperatures. 

### 3.5. Lyme Disease

There was a total of 1186 cases of nonfatal occupational injuries/illnesses involving days away from work due to the condition: Lyme disease, Table 2. The year 2015 saw a particularly high prevalence with 270 cases. Nevertheless, this assessment found no significant relationships based on temperature or time. 

### 3.6. Insecticides

There was a total of 5971 cases of nonfatal occupational injuries/illnesses involving days away from work due to the primary source: insecticides from 1992 to 2020, Table 2. There was a significant negative linear relationship between the frequency of non-fatal occupational injuries/illnesses due to insecticides and time (B = −13.718, *p* ≤ 0.001). National average temperatures were not predictive of the frequency of non-fatal occupational injuries/illnesses due to insecticides. When assessing these relationships based on the proportion of total annual incidents, there were no statistically significant relationships based on time or temperature.

### 3.7. Assumptions 

Regarding occupational fatalities due to exposure to environmental heat, both their frequency and their proportion of annual totals, and their relationship with time and annual average temperatures met all the assumptions, as did the assessment on fatal forest or brush fires and year, and nonfatal injuries/illnesses from insecticides and year, indicating good fitting models. However, the assessment of nonfatal forest fire injuries/illnesses over time had slightly heteroscedastic residuals. Nonfatal forest or brush fire injuries/illnesses and the average temperature had autocorrelated residuals with a Durbin Watson score of 1.4, slightly below the 1.5 cutoff, and residuals were slightly heteroscedastic. 

Assessments based on proportional data adjust for the decrease in total annual U.S. injuries and fatalities we can see in Table 1 and Table 2. However, they can also show a more obvious increase over time, which can indicate autocorrelation. It has been noted in time series analysis that autocorrelation may be viewed as a source of information about patterns of function and change rather than a statistical prohibition [22]. The proportion of nonfatal injuries/illnesses due to exposure to environmental heat and their relationship over time and average temperatures had residuals that were autocorrelated with Durbin Watson scores of 1.3 and 0.86, respectively. However, the autocorrelation is likely because the values on the time series are generally moving from smaller to larger, this steady increase is more obvious with the proportional data, thus resulting in some autocorrelation in the residuals. The proportion of nonfatal injuries due to forest or brush fires and their relationship with time and average temperature had slightly heteroscedastic residuals. The heteroscedasticity appears to be mainly caused by being on a time series where the dependent variable changes significantly over the time series. 

### 3.8. Summary

H1 can be partially accepted as the analysis found significant increases in occupational fatalities due to exposure to environmental heat when assessed by both their frequency and as a proportion of annual cases over the 30-year period. H2 can be partially accepted as there were significant increases in non-fatal environmental heat exposure cases when assessed as a proportion of total annual cases over the 29-year period. H3 can be partially accepted as national average annual temperatures had a significant positive relationship with fatalities due to exposure to environmental heat when assessed by both frequency and as a proportion of total cases. H4 can also be partially accepted as national average annual temperatures had a significant positive relationship with non-fatal occupational injuries involving days away from work due to exposure to environmental heat when assessed as a proportion of total cases.

## 4. Discussion

The national injury data over 30 years show occupational fatalities due to exposure to environmental heat are significantly increasing. There is a significant relationship between national average temperatures and occupational heat exposure fatalities when assessed by both the injury/illness frequency and as a proportion of annual totals. Occupational fatalities due to forest or brush fires are trending downward when assessed by their frequency. However, this relationship is not significant when assessing the proportion of these fatalities over annual totals. 

Nonfatal occupational injuries due to exposure to environmental heat are trending upward over the 29-year period and national average temperatures were predictive of heat exposure injuries/illnesses. However, this was only significant when assessed as a proportion of total annual injuries/illnesses. 

Nonfatal injuries/illness due to forest and brush fires are significantly trending downward over the 29-year period when assessed by both their frequency and proportion of annual totals. There was also a significant negative relationship between annual average temperatures and forest and brush fire-related injuries/illnesses, however, only when assessed by their frequency. Nonfatal injuries/illnesses due to insecticides did show a significant declining trend when assessed by their frequency. However, this relationship was not significant when evaluated as a proportion of total annual injuries. Finally, there were no significant national trends in occupational illnesses due to Lyme disease over the assessed period. 

There is evidence in the literature of the cause-and-effect nature of climate change, via high temperatures, and occupational health. A systematic review of the effects of climate change on workplace heat from individual and population-level studies in various regions around the globe confirms the heat injury association [2]. However, as noted by the authors, the majority of studies employed a weak design. Some studies focus on projected impacts based on modeling [23,24], while others examine cause-and-effect relationships with injury/illness data [25,26,27]. An assessment of Texas workers’ compensation injury cases and local temperatures specific to the day of occurrence found both high and low temperatures affected workers’ compensation claim rates. This study found high temperatures of 86–88 °F increase three-day claim rates by 2.1–2.8% and a day with temperatures over 100 °F increase claim rates by 3.5–3.7% [25]. An assessment of injury data in Alabama counties, over 5 years, found a significant increase in the number of heat-related occupational incidents over average summer temperatures [26]. However, it is difficult to link findings to climate change phenomena when looking at short periods of time as there are natural variations such as El Niño years, heatwaves, and other natural variations in climate and temperature that may influence results. Temperature averages associated with the effects of global warming change slowly. Assessing changes over longer periods, such as the 29-year and 30-year timeframe assessed in the present study allow for a more relevant comparison of trends that may be attributable to climate change.

During the period evaluated in the present study, most of the country has seen warmer temperatures between 1991 and 2020 according to National Oceanic and Atmospheric Administration’s National Center for Environmental Information’s U.S. Climate Normals [7]. While temperature averages change slowly, national and global assessment reports project dramatic and sustained rises in temperature for much of the United States over the next several decades [8,28]. It will be important to expand on this work in future studies that evaluate cases with state and local events and climatic conditions. Assessments of secondary effects of heat such as kidney disease, cardiovascular health, and the effect of cognitive functioning at work are also important. Further research can aid in building predictive models and the development of policies, interventions, and programs for occupational heat illness prevention and overall occupational injury prevention. 

A 2015 article examining how climate change is impacting wildland firefighters indicate wildland firefighter deaths are increasing due to climate change [29]. However, a 2017 assessment of wildland firefighter deaths notes limitations in classification and descriptions across multiple record-keeping systems which limit the ability to link cause and effect relationships needed to assess the occupational health burden [30]. The National Interagency Fire Center [31] tracks the frequency and extent of wildland fires. Their data show a general downward trend in the frequency of wildland fires and an increase in the extent of the fires by the number of acres burned [31,32]. Thus, though less frequent, the extent of the fires is increasing over time. Despite larger fires, data presented in this study show worker forest and brush fire deaths and injuries are decreasing which is possibly an attestation to sound occupational safety management in the fire service and first responder adoption of safety interventions from training, following safe practices, use of personal protective equipment and better engineering to reduce the likelihood and severity of worker harm. However, further research is required to confirm the effectiveness of safety interventions and injury prevention and the burden of wildland fires on occupational health. 

There are no significant trends, at the national level, of Lyme disease as a condition in which workers are missing time off of work due to their illness. This finding is supported in the literature as the effects of climate change on vector-borne disease and occupational health are scarce [2]. However, there is literature on the increasing prevalence of Lyme disease in the general population in the Northeastern U.S. and Canada [33,34,35]. As noted previously, because temperature, humidity and precipitation are important factors in how humans are exposed to blacked-legged ticks that carry Lyme disease [14,15] an assessment at the regional and state level based on time, temperature, humidity, and precipitation is warranted.

Insecticides are indicated as a potential worker health impact of climate change as more and different insecticides may be used on crops and many workers may opt to use insecticides to protect themselves from the dangers of vector-borne disease [16]. However, the review of 29-year historical data indicates this is currently not a growing problem for the health and safety of American workers. Nevertheless, this trend may be more noticeable at the state or regional levels and thus more localized assessments are recommended.

### 4.1. Practical Implications for Occupational Health and Safety Management

The results of this study highlight the increasing trends in occupational illnesses and fatalities due to exposure to environmental heat. Occupational heat exposure management will become increasingly important in policy and planning for safe work in the United States, particularly in the summer months when workers are exposed to elevated temperatures. Heat is the leading cause of death among all weather-related workplace hazards [36]. Program and personal interventions such as those recommended in the National Institute for Occupational Safety and Health (NIOSH) Criteria for a Recommended Standard: Occupational Exposure to Heat and Hot Environments [37], and NIOSH Heat Stress [38] should be adopted by organizations to aid in heat illness prevention. While some states including California, Minnesota, and Washington have their own heat illness prevention standard or rule under their state-run plans, OSHA, at the national level, has not implemented a workplace rule on heat exposure. However, it has been proposed and is in process as of 2021 [36]. 

Measurement of heat exposure from external sources and work activities is an important factor in designing a heat stress program. Sabrin et al. [26] suggest the use of a modified index using a combination of a heat balance model and a physiological index that captures workers’ heat perception along with their physiological strain level. Individual strain levels would be mainly characterized by the use of wearables as a means to adjust for any effects of thermoregulating properties from clothing and individual pre-conditions that influence how one responds to heat and work. However, how this index can be easily operationalized is forthcoming from the authors. NIOSH Criteria for a Recommended Standard: Occupational Exposure to Heat and Hot Environments [37] considers these aspects as well as individual age and physical condition and makes recommendations for policy, programs, engineering controls, and personal protective equipment to minimize the effects of heat. 

Managing heat stress has implications for occupational health and safety management beyond heat-related illness. A Chinese study found that heat increase resulted in increased occupational injury claims beyond the scope of heat-related illness [39]. This finding is not surprising as environmental heat is a major topic of concern in occupational ergonomics and human factors engineering as it is known to cause fatigue and reduced mental processing [40] which can lead to human errors and mishaps [41]. 

While wildland fires are becoming larger in terms of acreage burned, this is not yet affecting worker fatality and injury data recorded by the CFOI and SOII. However, as noted above, there are other data sources and conflicting information on classifications that make defining the occupational health burden from wildland fires difficult [30]. Nevertheless, first responders, forestry workers, and anyone working within these environments should remain vigilant and follow all safety protocols including managing heat stress and use of respirators for protection against smoke. The National Wildfire Coordinating Group has published the Wildland Fire Incidents Management Field Guide [42] and A Preparedness Guide for Wildland Firefighters and Their Families [43] which cover safety protocols. Guidance on wildfire safety such as that provided by the National Fire Protection Agency [44] and the American Red Cross [45] is suitable for non-first responders and the general public.

While human cases of Lyme disease and other tick-borne diseases reported each year to the CDC have been increasing steadily in the United States [46], the injury data do not yet reflect a significant increasing trend on the national scale. However, this may not be true at the state and local levels. For industries and occupations that work in outdoor areas where ticks are prevalent, the EPA has several resources and initiatives to address tick populations [47] and to aid in planning safe work including guidance on insect repellants and tick-repellent-treated clothing [46]. 

### 4.2. Limitations

While this study contributes to the literature on occupational health and safety and the impacts of climate change, it is not without limitations. There was heteroscedasticity in the residuals of non-fatal forest fire injuries/illnesses over time and temperature, and the proportion of non-fatal injuries due to forest or brush fires and their relationship with time and average temperature. Non-fatal forest or brush fire injuries/illnesses and average temperature and the proportion of non-fatal injuries/illnesses due to exposure to environmental heat and their relationship over time and average temperatures had residuals that were autocorrelated. While the heteroscedasticity and the autocorrelations are likely because the values on the time series are generally moving from smaller to larger, this increase is more obvious with the proportional data, thus resulting in some autocorrelation in the residuals. The interpretation of the coefficients of these regressions may be less accurate.

Injury and fatality data are cross-sectional which limits causal inferences. While the scope of this assessment was to assess occupational injury/illness data that may be related to the impacts of climate change, there were limitations to the availability of data. Data on non-fatal injuries for all ownership types were not available for the 29-year period as BLS has only recorded this information since 2008. This may limit the generalizability of the results across all ownership types. 

Assessing injury and fatality data at the national level also limits the ability to infer climate change impacts as causal factors. Case-by-case assessment of injuries and fatalities would be needed to assess the date, time, location and the scenarios leading up to the injury/fatality in order to compare with local temporal, environmental, and climatic conditions. However, access to CFOI and SOII metadata is limited. Nevertheless, this research contributes to the literature on assessing the historical burden of projected climate change-related impacts on occupational health and serves as a starting point on which to build future studies at the regional, state, and local levels. Future assessments specific to industry and occupations that consider more germane and granular information can help support or negate any cause-and-effect relationships between climate change and the effect on occupational health and safety. 

## 5. Conclusions

The purpose of this study was to assess for significantly increasing national trends in the prevalence of occupational injuries/illnesses and fatalities related to heat exposure, wildfires, Lyme disease, and pesticides, and their relationship with national temperature trends, as these illnesses have been indicated as projected climate change impacts on occupational health. The only fatalities and illnesses assessed that have significantly increasing trends over time were due to exposure to environmental heat. Annual average temperatures were significant predictors of their increase. Heat exposure is an occupational hazard that must be managed carefully in the coming years. Organizations will need to take more aggressive heat exposure control measures as temperatures continue to rise and remain hotter for longer periods of the year. While not currently showing increasing trends, the prevalence of incidents due to forest or brush fires, Lyme disease, and insecticides should be investigated further as the United States experiences more of the projected impacts of climate change. 

While these assessments were made on a national level using linear models, they reveal historical trends over the last 29–30 years and can be a catalyst for localized assessments that can aid in creating more sophisticated models. Future research based on robust data can support policymakers, employers, and occupational health and safety professionals in assessing risks associated with climate change and support planning for injury and illness prevention.

## Figures and Tables

**Table 1:** All U.S.—all ownerships—fatal occupational injuries and illnesses totals and frequency and percentage due to exposure to environmental heat and forest or brush fires and national average temperatures °F 1992–2021.

Year	Total U.S.	Env. Heat	%	Forest or Brush Fire	%	Avg. Temp. °F
1992	6217	12	0.19	5	0.08	52.6
1993	6331	22	0.35	4	0.06	51.26
1994	6632	28	0.42	20	0.30	52.87
1995	6275	35	0.56	9	0.14	52.65
1996	6202	18	0.29	10	0.16	51.89
1997	6238	22	0.35	8	0.13	52.2
1998	6055	34	0.56	14	0.23	54.23
1999	6054	35	0.58	6	0.10	53.88
2000	5920	21	0.35	6	0.10	53.27
2001	5915	24	0.41	7	0.12	53.7
2002	5534	40	0.72	9	0.16	53.21
2003	5575	29	0.52	9	0.16	53.26
2004	5764	18	0.31	7	0.12	53.1
2005	5734	47	0.82	10	0.17	53.64
2006	5840	44	0.75	11	0.19	54.25
2007	5657	32	0.57	6	0.11	53.65
2008	5214	27	0.52	9	0.17	52.29
2009	4551	35	0.77	5	0.11	52.39
2010	4690	40	0.85	4	0.09	52.98
2011	4693	61	1.30	12	0.26	53.18
2012	4628	31	0.67	0	0.00	55.28
2013	4585	34	0.74	22	0.48	52.43
2014	4821	18	0.37	3	0.06	52.54
2015	4836	37	0.77	4	0.08	54.4
2016	5190	39	0.75	3	0.06	54.92
2017	5147	32	0.62	7	0.14	54.55
2018	5250	49	0.93	6	0.11	53.52
2019	5333	43	0.81	0	0.00	52.68
2020	4764	56	1.18	0	0.00	54.37
2021	5190	36	0.69	0	0.00	54.51
Total	164,835	999	0.61	216	0.13	

**Table 2:** All U.S.—private ownership—non-fatal occupational injuries/illnesses totals and frequency and percentage due to exposure to environmental heat, forest or brush fire, Lyme disease, and insecticides 1992–2020.

Year	Total U.S.	Env. Heat	%	Forest or Brush Fire	%	Lyme Disease	%	Insect.	%
1992	2,331,098	3629	0.16	163	0.01	0	0.000	589	0.025
1993	2,252,591	3119	0.14	92	0.00	32	0.001	462	0.021
1994	2,236,639	2540	0.11	94	0.00	16	0.001	493	0.022
1995	2,040,929	3629	0.18	52	0.00	74	0.004	249	0.012
1996	1,880,525	1789	0.10	53	0.00	59	0.003	466	0.025
1997	1,833,380	1630	0.09	0	0.00	0	0.000	200	0.011
1998	1,730,534	2738	0.16	38	0.00	69	0.004	236	0.014
1999	1,702,470	2708	0.16	0	0.00	0	0.000	104	0.006
2000	1,664,018	2554	0.15	127	0.01	79	0.005	382	0.023
2001	1,537,567	3135	0.20	18	0.00	0	0.000	482	0.031
2002	1,436,194	2666	0.19	78	0.01	17	0.001	118	0.008
2003	1,315,920	2060	0.16	60	0.00	70	0.005	210	0.016
2004	1,259,320	1590	0.13	20	0.00	110	0.009	180	0.014
2005	1,234,680	2610	0.21	0	0.00	40	0.003	140	0.011
2006	1,183,500	3110	0.26	50	0.00	20	0.002	300	0.025
2007	1,158,870	2550	0.22	50	0.00	90	0.008	70	0.006
2008	1,078,140	1660	0.15	100	0.01	30	0.003	70	0.006
2009	964,990	1790	0.19	20	0.00	30	0.003	120	0.012
2010	933,200	3470	0.37	0	0.00	130	0.014	50	0.005
2011	918,140	3400	0.37	0	0.00	0	0.000	130	0.014
2012	918,720	3310	0.36	0	0.00	0	0.000	100	0.011
2013	917,090	2550	0.28	0	0.00	0	0.000	60	0.007
2014	916,440	2070	0.23	0	0.00	0	0.000	30	0.003
2015	902,160	2010	0.22	0	0.00	270	0.030	70	0.008
2016	892,270	3300	0.37	0	0.00	0	0.000	450	0.050
2017	882,730	2490	0.28	0	0.00	30	0.003	50	0.006
2018	900,380	3120	0.35	0	0.00	0	0.000	60	0.007
2019	888,220	2410	0.27	0	0.00	20	0.002	40	0.005
2020	176,340	1940	1.10	0	0.00	0	0.000	60	0.034
Total	38,087,055	75,577	0.20	1015	0.003	1186	0.003	5971	0.016

**Table 3:** Regression results based on frequency of annual incidents.

No. of Incidents		B	Std. Error	β	*t*	*p*	R	R^2^	Adj. R^2^	F
***Fatal occupational injuries/illnesses***										
Env. heat exposure	Year	0.723	0.205	0.554	3.524	0.001 **	0.554	0.307	0.282	12.415
	Avg. temp	4.912	2.064	0.410	2.380	0.024 *	0.410	0.168	0.139	5.663
Forest or brush fires	Year	−0.242	0.102	−0.410	−0.410	0.024 *	0.410	0.168	0.139	5.670
	Avg. temp	−1.501	0.985	−0.277	−1.523	0.139	0.277	0.076	0.044	2.319
***Non-fatal occupational injuries/illnesses***										
Env. heat exposure	Year	−8.869	14.194	−0.119	−0.625	0.537	0.119	0.014	−0.022	0.390
	Avg. temp	155.730	124.777	0.234	1.248	0.223	0.234	0.055	0.020	1.558
Forest or brush fires	Year	−3.491	0.770	−0.657	−4.533	<0.001 **	0.657	0.432	0.411	20.547
	Avg. temp	−18.328	8.468	−3.85	−2.164	0.039 *	0.385	0.148	0.116	4.685
Lyme disease	Year	0.100	1.303	0.015	0.077	0.939	0.015	0.000	−0.037	0.006
	Avg. temp	5.552	11.645	0.091	0.477	0.637	0.091	0.008	−0.028	0.227
Insecticides	Year	−13.718	2.810	−0.685	−4.882	<0.001 **	0.685	0.469	0.449	23.834
	Avg. temp	−35.914	33.910	−0.200	−1.059	0.299	0.200	0.040	0.004	1.122

* Significant at the 0.05 level. ** Significant at the 0.001 level.

**Table 4:** Regression results based on proportion of annual incidents.

Proportion of Annual Incidents		B	Std. Error	β	*t*	*p*	R	R^2^	Adj. R^2^	F
***Fatal occupational injuries/illnesses***										
Env. heat exposure	Year	0.00019	0.00004	0.665	4.72	<0.001 **	0.665	0.443	0.423	22.229
	Avg. Temp	0.001	0.00005	0.397	2.29	0.030 *	0.397	0.157	0.127	5.232
Forest or brush fire	Year	−2.985 × 10^−5^	0.00002	−0.267	−1.47	0.153	0.267	0.071	0.038	2.156
	Avg. temp	−0.000277	0.00019	−0.270	−1.48	0.149	0.270	0.073	0.040	2.204
***Non-fatal occupational injuries/illnesses***										
Env. heat exposure	Year	0.000134	0.00003	0.616	4.07	<0.001 **	0.616	0.380	0.357	16.540
	Avg. temp	0.001	0.00034	0.427	2.45	0.021 *	0.427	0.182	0.152	6.022
Forest or brush fire	Year	−1.657 × 10^−6^	5.077 × 10^−7^	−0.522	−3.18	0.004 *	0.522	0.273	0.246	10.122
	Avg. temp	−9.522 × 10^−6^	0.000005	−0.334	−1.84	0.076	0.334	0.112	0.079	3.398
Lyme disease	Year	1.056 × 10^−6^	0.000001	0.149	0.784	0.440	0.149	0.022	−0.014	0.615
	Avg. temp	9.680 × 10^−6^	0.000012	0.152	0.802	0.430	0.152	0.023	−0.013	0.642
Insecticides	Year	−1.945 × 10^−6^	0.000002	−0.153	−0.802	0.429	0.153	0.023	−0.013	0.643
	Avg. temp	2.470 × 10^−5^	0.000022	0.216	1.15	0.261	0.216	0.047	0.011	1.319

* Significant at the 0.05 level. ** Significant at the 0.001 level.

## Data Availability

Data were collected on fatal and non-fatal occupational injuries from the United States Bureau of Labor Statistics (BLS) online profiles tool and National Oceanic and Atmospheric Administration (NOAA) National Centers for Environmental Information, Climate at a Glance: National Time Series.
